# Applicability of electronic health records in the hospital Nursing Process: a scoping review

**DOI:** 10.1590/0034-7167-2024-0520

**Published:** 2025-12-08

**Authors:** Isabela Rodrigues da Silva, Elisana Agatha Iakmiu Camargo Cabulon, Fernanda Santiago Santos Mendonça, Flavia Mendonça da Silva Oussaki, Maria do Carmo Fernandez Lourenço Haddad

**Affiliations:** I Universidade Estadual de Londrina. Londrina, Paraná, Brazil; II Universidade Estadual de Maringá. Maringá, Paraná, Brazil; III Universidade Federal de São Paulo. São Paulo, São Paulo, Brazil

**Keywords:** Nursing Process, Nursing Care, Nursing Informatics, Electronic Health Records, Electronic Medical Records., Proceso de Enfermería, Atención de Enfermería, Informática Aplicada a la Enfermería, Registros Electrónicos de Salud, Registros Médicos.

## Abstract

**Objectives:**

to synthesize scientific evidence on the applicability of Electronic Health Records in the Nursing Process in hospitals.

**Methods:**

a scoping review following the Preferred Reporting Items for Systematic Reviews and Meta-Analyses guidelines, with searches in six electronic databases, restricted to publications in Portuguese, English, and Spanish from 2019 to 2024.

**Results:**

a total of 2,789 results were found, with 33 articles included in the search and classified into four thematic categories. The countries with the most publications include Brazil, the United States, and China, with a focus on 2021.

**Conclusions:**

hospital Nursing Process records are crucial for verifying care and managing risks, improving quality and patient safety. Implementing Electronic Health Records optimizes work and reduces costs, alleviating staff burden. In addition to supporting clinical decision-making, these records also generate secondary data that drives scientific research.

## RESUMEN

**Objetivo:**

sintetizar la evidencia científica sobre la aplicabilidad de los Registros Electrónicos de Salud en el proceso de enfermería hospitalario.

**Métodos:**

revisión de alcance siguiendo las directrices del *Preferred Reporting Items for Systematic Reviews and Meta-Analyses*, con búsquedas en seis bases de datos electrónicas, restringidas a publicaciones en portugués, inglés y español, entre 2019 y 2024.

**Resultados:**

se encontraron 2789 resultados, con 33 artículos incluidos en la búsqueda, clasificados en cuatro categorías temáticas. Los países con más publicaciones son Brasil, Estados Unidos y China, con especial atención a 2021.

**Conclusiones:**

los registros del Proceso de Enfermería Hospitalaria son cruciales para verificar la atención y gestionar los riesgos, mejorando la calidad y la seguridad del paciente. La implementación de Registros Electrónicos de Salud optimiza el trabajo y reduce los costos, aliviando la carga del personal. Además de respaldar la toma de decisiones clínicas, estos registros también generan datos secundarios que impulsan la investigación científica.

## INTRODUCTION

**Figure 1:**
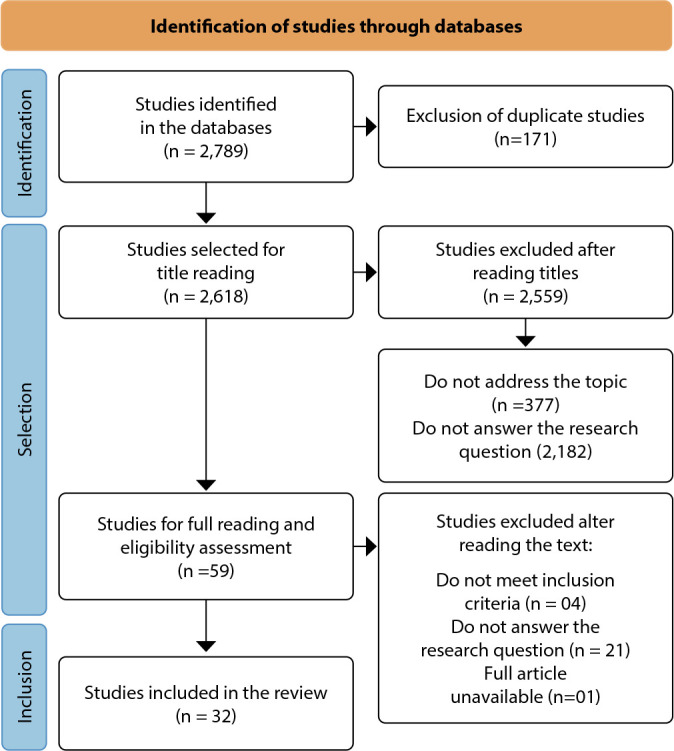
Flowchart of the study selection process according to the Preferred Reporting Items for Systematic Reviews and Meta-Analyses for Scoping Reviews guidelines

Introduced in Brazil by Wanda Horta, the Nursing Process (NP) concept allowed the care provided to individuals to acquire a formalized and systematized character^(1)^. This concept concerns the organization of nurses’ work, in order to guarantee quality in the care provided^(2)^. In this context, technological innovations, such as Electronic Health Records (EHRs), emerge as strategic tools to enhance NP operationalization in healthcare institutions^(3)^.

In Brazil, the implementation of digital systems for managing health information has advanced over the years. Beginning in 2013, with initiatives such as the Brazilian National Primary Care Policy and the Healthcare Network, the use of computerized systems, such as the Electronic Patient Record (EPR), began to expand, aiming to improve quality of care and clinical information management^(4)^.

In 2024, the Federal Nursing Council published Resolution 736/2024, mandating the NP implementation in all socio-environmental contexts in which nursing care occurs. Due to nursing’s new perspectives, delimiting its scope of application in practice is necessary, adapting NP as a tool to guide nurses’ clinical reasoning in the work process^(5)^.

As this is legal and ethical documentation of nursing practice, it is essential to formally record NP in patients’ medical record, whether physical or electronic^(5)^. From this perspective, the digital transformation in healthcare services has promoted significant changes in nursing practice, especially with the implementation of EHRs in hospital settings^(6)^.

Studies show that without NP records, it is not possible to systematically recover the work process performed by nursing, which reinforces effective strategies to improve quality of clinical records in healthcare services^(2)^. Such strategies often include innovations in the field of communication and information, which play a fundamental role in improving data capture and improving the care provided^(7)^.

In this regard, technologies such as EHR aim to optimize the capture, access and sharing of clinical information, directly impacting care quality and safety^(8)^. The use of EHR facilitates rapid access to updated patient data, supports informed clinical decision-making, promotes standardization and readability of records, and contributes significantly to the efficiency and safety of care provided^(6)^.

A study showed that more than half of technologies supporting EHRs were related to the development of software aimed at teaching and learning nurses^(9)^. In general, such technologies allow for better document control and archiving of information related to care, contributing to the improvement of work processes and qualifying the care provided to patients^(10)^.

Specifically in hospital settings, the use of EPR as an EHR and technological communication tool stands out, the purpose of which is to manage efficiency and effectiveness in hospital management^(11)^. EPR is a secure strategy for recording and storing patient information that can be shared among the healthcare team, preventing adverse events^(12)^.

However, the use of EPR in NP faces several weaknesses that impact quality of care and the efficiency of professionals’ work^(8)^. Resistance to change among healthcare professionals, combined with a lack of adequate training, makes it difficult to adapt to the system, resulting in inaccurate or incomplete records^(13)^. Furthermore, data security and privacy are also a constant concern, as improper use of EPR can expose sensitive information to cyber risks^(14)^.

Therefore, it is essential to investigate and propose solutions that strengthen NP practice, promoting its integration into nurses’ work routines and improving quality of clinical records, which is fundamental for patient safety and efficiency in hospital management^(15)^.

## OBJECTIVES

To synthesize scientific evidence on the applicability of EHR in NP in a hospital setting and how these findings can contribute to nursing practice.

## METHODS

### Study design, period and location

This is a scoping review based on the JBI scoping review manual theoretical and methodological framework, following the stages outlined in the Preferred Reporting Items for Systematic Reviews and Meta-Analyses for Scoping Reviews checklist. In addition to tracking studies on the topic of interest, this type of research examines the extent and potential of the findings to become systematic reviews^(16)^. The protocol for this review was previously registered on the Open Science Framework platform at the address: https://osf.io/86bpn/.

A temporal cut was made from 2019 to 2024 and language, including studies in Portuguese, English and Spanish, due to the large number of results, although the JBI methodological recommendation is more comprehensive.

To reach diverse types of studies, the following electronic databases were used: Medical Literature Analysis and Retrieval System Online (MEDLINE via PubMed); Latin American and Caribbean Health Sciences Literature (LILACS); Web of Science (WoS); Excerpta Medica dataBASE (Embase); Elsevier (Scopus); and Cumulative Index to Nursing and Allied Health Literature (CINAHL). Duplicate studies and those not fully accessible in open or restricted databases were not included in this study.

In line with the methodological assumptions of scoping reviews, we prioritized the scope and relevance of studies to the research question-the applicability of EHRs in NP in a hospital setting-regardless of individual design or methodological rigor. This approach enabled a broad and descriptive analysis of existing literature, appropriate to the exploratory objectives of the review.

### Population or sample; inclusion and exclusion criteria

The Population, Concept, and Context (PCC) strategy was employed, with P being the applicability of EPR in the development of NP, C being the number and content of published scientific evidence on the use of EPR in NP, and C being hospital settings. From this, the following research question emerged: what scientific evidence is there for the applicability of EHR in NP in a hospital setting? It was possible to map and explore the scientific literature on the chosen topic, allowing us to identify how studies were conducted^(8)^.

The studies included experimental, quasi-experimental (randomized and non-randomized clinical trials), observational (cohort, case-control, and cross-sectional), and qualitative designs. This review also included case reports, protocols, clinical practice guidelines, all types of evidence syntheses, theses, dissertations, and undergraduate thesis papers.

Articles with incomplete data, letters, editorials and studies that, after three consecutive attempts to contact the authors, were not available in full were not included in this scoping review.

### Study protocol

The search strategy was adapted for each database according to the descriptors recommended in each. The terms were combined with the Boolean operators “AND” and “OR” in the Virtual Health Library and Embase databases to identify search terms related to the research topic through studies considered relevant. After using the Health Sciences Descriptors (DeCS) and Medical Subject Headings (MeSH) platforms, Chart 1 described the strategies used in each database.

**Chart 1:** Search strategies on the Health Sciences Descriptors and Medical Subject Headings platforms, 2024

Database	Type	Search strategies
MEDLINE (via PubMed)	MeSH	(((“Nursing Process”) OR (“Nursing Care”)) AND (“Nursing Informatics”)) OR (“Electronic Health Records”)
WoS	MeSH	(“Nursing Process”) OR (“Patient Care Planning”) AND (“Nursing Informatics”) OR (“Electronic Health Records”)
Scopus	MeSH	(“Nursing Process”) OR (“Patient Care Planning”) AND (“Nursing Informatics”) OR (“Electronic Health Records”)
LILACS	DeCS	(“*Processo de Enfermagem*”) OR (“*Cuidados de Enfermagem*”) AND (“*Informática em Enfermagem*”) OR (“*Registros Eletrônicos de Saúde*”)
Embase	*Emtree* *Thesaurus*	(‘Nursing Process’ OR ‘Nursing Care’) AND (‘Nursing Informatics’ OR ‘Medical Record’)
CINAHL	MeSH	(“Nursing Process” OR “Nursing Care”) AND (“Nursing Informatics” OR “Nursing Records” OR “Electronic Health Record”)

*WoS - Web of Science; DeCS - Health Sciences Descriptors; MeSH - Medical Subject Headings; LILACS - Latin American and Caribbean Literature in Health Sciences; MEDLINE - Medical Literature Analysis and Retrieval System Online; CINAHL - Cumulative Index to Nursing and Allied Health Literature; Embase - Excerpta Medica dataBASE.*

### Analysis of results and statistics

The studies were grouped and exported to the Rayyan QCRI platform, enabling blind and impartial screening and selection between two independent reviewers, after automatically excluding duplicate studies. This tool allowed the screening to be performed without the reviewers having access to each other’s decisions, ensuring the neutrality of the process. In cases of disagreement between the reviewers regarding study inclusion or exclusion, a third reviewer was called in to resolve the impasse.

Initial analysis was conducted by reading the titles and abstracts, identifying those that aligned with the research question. The selected studies were then read in full to confirm their eligibility, and those that met the established criteria were summarized descriptively, organized into tables, and accompanied by a narrative summary, according to the methodological stages recommended by the JBI scoping review manual.

Still in accordance with JBI recommendations, data mapping occurred with data extraction, including database, authorship, country, year, objective and applicability of EHR in NP. This information was fed into a Microsoft Office Excel^®^ 2019 spreadsheet, enabling data extraction for the synthesis.

As a result of study selection, a flowchart was created to describe all the stages and their respective results. Furthermore, the data were organized into descriptive tables to facilitate understanding and contribute to the discussion of results.

Along with tabulating the results, the *Interface de R pour les Analyses Multidimensionnelles de Textes et de Questionnaires* (IRAMUTEQ) software was used to group the results, generating a dendrogram with classes to highlight relevant situations and similarities between findings. This enabled a narrative description of the findings and directed correlation with the review’s objective and question.

## RESULTS

A total of 2,789 publications were found, of which 171 were excluded due to duplication, leaving 2,618 studies for reading of titles and abstracts, of which 59 were selected for full-text reading. Of these, 33 were included in the review because they answered the research question and met the eligibility criteria.

Of the total articles included in this scoping review, 13 were published in Brazil, four in the United States, four in China, two in Finland, two in Turkey, and two in Australia. Iran, Switzerland, Canada, Mexico, and Italy each published one article.

The IRAMUTEQ software was used to analyze similarity among the included studies, providing four classes according to the statistics of the program’s text *corpus*, with a 75% utilization rate.

From the *corpus*, the text segments presented in each class were obtained from statistically significant words, allowing qualitative data analysis. *Corpus* processing took seven seconds, resulting in 966 forms (*Unité Contextuelle d’Enregistrement*) being classified, of which 698 were used, resulting in 75% of the total *corpus*, considered adequate.

By reading the highlighted words and inserting them into text segments, we were able to achieve the research objectives of identifying scientific evidence for the applicability of EHR in NP in hospital settings. Charts 2, 3, 4, and 5 below present a summary of information regarding the studies included in this scoping review, categorized according to the classes organized by IRAMUTEQ.

**Chart 2:** Summary of articles included in class 1 of the *Interface de R pour les Analyses Multidimensionnelles de Textes et de Questionnaires* for the final sample of the scoping review, Londrina, Paraná, Brazil, 2024

Database, first author, country and year	Study design	Objectives	Applicability of EHR in NP
MEDLINE, Karp *et al*., USA, 2019^(17)^	Prospective cohort study	Assess whether EPR system timers and event logs can measure the efficiency and quality of a clinical process.	Efficiency and quality of the clinical process by optimizing the time spent on nursing records.
CINAHL, Ozduyan Kilic and Korkmaz Turkey, 2023^(18)^	Literature review	Assess the extent to which nurses in Türkiye use EHR systems/hospital information systems and what data are recorded.	Nursing data related to medical treatment were documented more frequently electronically, while those related to the nursing process were maintained on paper.
CINAHL, Sanchez *et al*., Brazil, 2019^(19)^	Qualitative descriptive study, case study design	Assess nurses’ records in electronic and conventional patient records, with a view to improving quality of care.	Improved coordination, quality of care, and patient safety.
CINAHL, Menezes and Lopes Neto, Brazil, 2019^(20)^	Technological production study, qualitative approach	Report the development of software for Systematization of Nursing Care in tropical and infectious diseases.	The use of the software improved nursing care performance, team communication, and agility in decision-making.
CINAHL, Faruch *et al*., Brazil, 2021^(21)^	Cross-sectional, retrospective, documentary study, with a quantitative approach to data	Assess NP implementation in a university hospital.	The use of software facilitates the implementation of the nursing process, providing support by providing data for assessing diagnoses, interventions, and recording relevant information about staff and patients.
MEDLINE, Macieira *et al*., USA, 2019^(22)^	Systematic review	Present the results of a systematic review of studies involving secondary analyses of data coded with standardized nursing terminologies retrieved from EHRs.	EHR improves efficiency, standardizes nursing care documentation, generates consistent data, facilitates information sharing, and enables analysis to improve clinical practice and patient outcomes.
MEDLINE, Yilmaztturk *et al*., Turkey, 2023^(23)^	Observational study	Measure the effect of digitizing medical records kept on paper in the ICU on saving time and paper consumption.	This saves time and money in hospital settings.
Scopus, Fritzen *et al*., Brazil, 2023^(24)^	Experimental study	Implement, in health management software, electronic records of perioperative NP and the stages of the intraoperative and immediate postoperative period, in addition to nursing diagnoses based on NANDA-I.	Electronic records of perioperative NP enable health management, contributing to the improvement of care practices.
Embase, Chen *et al*., China, 2021^(25)^	Retrospective chart review	Investigate the clinical characteristics of constipation and its risk factors in patients with intracerebral hemorrhage using electronic nursing care records.	EHR resulted in the identification of patients with constipation.

*EHR - Electronic Health Record; EPR - Electronic Patient Record; PE - Nursing Process; ICU - Intensive Care Unit; NANDA-I - NANDA International; USA - United States of America; Embase; MEDLINE - Medical Literature Analysis and Retrieval System Online; CINAHL - Cumulative Index to Nursing and Allied Health Literature; Embase - Excerpta Medica dataBASE.*

**Chart 3:** Summary of articles included in class 2 of the *Interface de R pour les Analyses Multidimensionnelles de Textes et de Questionnaires* for the final sample of the scoping review, Londrina, Paraná, Brazil, 2024

Database, first author, country and year	Study design	Objectives	Applicability of EHR in NP
CINAHL, Zhao *et al*., China, 2021^(26)^	Case-control study	Develop a new system to improve nursing practice and ensure continuity and integrity in hyperthermia nursing care.	The developed system assessed the improvement in quality of nursing care, optimizing care time and system experience for nurses, and improving quality of nursing records.
CINAHL, Oliveira *et al*., Brazil, 2021^(27)^	Quasi-experimental quantitative study	Compare quality of NP documentation in two versions of a clinical decision support system.	Integration with clinical decision support systems facilitates analysis and support in nursing professionals’ decision-making, in addition to contributing to the organization of information, ensuring continuity and quality of care provided to patients.
CINAHL, *et al*., Brazil, 2019^(12)^	Methodological study	Describe the construction and validation of electronic NP records in an oncology institution.	It favors nurses’ decision-making and care qualification through interconnected electronic records.
MEDLINE, Akbar *et al*., Australia, 2021^(28)^	Systematic review	Summarize the literature on automated decision support systems in nursing and analyze their effects on care delivery and patient outcomes.	Integration of decision support systems, adherence to information and documentation of nursing interventions promote efficiency and quality in healthcare.
Scopus, Von Gerich *et al*., Finland, 2023^(29)^	Pilot study	Explore the potential of EHRs as a source of information to assess the effectiveness of nursing care.	Electronic records are sources of information in the development of clinical decision support systems, health management and assessment of the effectiveness of nursing care.
Scopus, Franco *et al*., Brazil, 2023^(30)^	Experience report	Describe the development of the computerization of risk prediction scales, used by nursing in electronic medical records, in the AGHUse^®^ system.	The functionalities developed in the computerization of risk prediction scales favored their operationalization, reflecting positively on nursing practice and patient safety.
Scopus, Hants *et al*., Australia, 2023^(31)^	Integrative systematic review	Identify how NP was incorporated into digital health records.	The computerization of all stages of NP enables the advancement of nurses’ decision-making processes, as well as the visibility of their work.
Scopus, Szydłowska-Pawlak *et al.,* Switzerland, 2022^(32)^	Case study	Formulate a care plan for a newborn diagnosed with congenital gastroschisis in the postoperative period, using the International Classification for Nursing Practice, which was included in the “ADPIECare Dorothea” software.	EHRs are tools that support nurses’ clinical decision-making. The data can help optimize work organization to achieve high levels of patient health and safety.
Scopus, Wu *et al*., China, 2020^(33)^	Review study	Describe the challenges of EHRs and the application of an NP-based nursing decision support system.	The clinical decision support system is supported by the electronic records that nurses collect during care, which allows for improved quality and safety of care.
Scopus, Bail *et al.,* Australia, 2021^(34)^	Retrospective audit	Explore and compare NP documentation for patient safety in two nursing documentation systems: paper and digital records.	Electronic systems have the potential to improve the capture of clinical reasoning and NP.
Scopus, Sanson *et al.,* Italy, 2019^(35)^	Prospective observational study	Explore the independent predictive power of nursing diagnoses on in-hospital mortality and investigate whether including this variable in addition to medical diagnosis data can improve the performance of risk adjustment tools.	Nursing diagnoses have independent power in predicting in-hospital mortality. The variance explained in predictive models improved when electronic nursing data were included in addition to medical data.
MEDLINE, Burkoski *et al*., Canada, 2021^(36)^	Descriptive study	Describe the development of Humber River Hospital’s innovative electronic medical record-based approach to triaging patients at risk for adverse outcomes, using the Ontario Association of Registered Nurses and best practice guidelines.	Electronic records facilitate assessments and interventions to provide evidence-based care. They also prevent nurses from aligning their actions with established standards, reducing variability in nursing practice.
LILACS, Chen Weiwin *et al.,* Mexico, 2021^(37)^	Cross-sectional, retrospective, documentary study	Assess compliance with the electronic clinical record according to the nursing care model.	Nursing records are essential for evidence-based care decision-making. They allow for the identification of areas of opportunity for improvement, reinforcing adherence to standardized nursing record models.
LILACS, Schamne *et al*., Brazil, 2019^(38)^	Mixed-method, exploratory, descriptive study	Validate a proposal for nursing care records containing diagnoses, expected outcomes, current outcomes, interventions, and nursing actions for patients with clinical deterioration, using the Clinical Care Classification System.	It contributes to improving nurses’ decision-making and improving information flows regarding the care provided to patients with clinical deterioration.

*EHR - Electronic Health Record; NP - Nursing Process; LILACS - Latin American and Caribbean Literature in Health Sciences; MEDLINE - Medical Literature Analysis and Retrieval System Online; CINAHL - Cumulative Index to Nursing and Allied Health Literature.*

**Chart 4:** Summary of articles included in class 3 of the *Interface de R pour les Analyses Multidimensionnelles de Textes et de Questionnaires* for the final sample of the scoping review, Londrina, Paraná, Brazil, 2024

Database, first author, country and year	Study design	Objectives	Applicability of EHR in NP
MEDLINE, Kutney-Lee *et al*., USA, 2019^(39)^	Cohort study	Examine the effects of comprehensive EHR adoption on nurses’ reports on quality of care.	Quality of patient care and contribution to patient safety.
Scopus, Shafiee *et al*., Iran, 2022^(40)^	Methodological research	Describe the process of designing and assessing the content of an electronic nursing clinical documentation system to provide consistent and unified reports.	The electronic system reduces the amount of data collected during nursing care, resulting in greater job satisfaction due to the reduced workload associated with this activity.
LILACS, Costa *et al*., Brazil, 2020^(41)^	Integrative review	Identify the strategies being used in the implementation of electronic nursing records related to nursing procedures in the PubMed, Scopus, and Web of Science databases.	Computerized systems are widely adopted in healthcare, requiring nurses to be trained to standardize nursing records, which requires constant improvement.
LILACS, Amaral *et al*., Brazil, 2021^(42)^	Cross-sectional study	Explore nurses’ opinions on the electronic recording of nursing diagnoses and disciplines in a computerized system within the hospital setting.	Computerization generates a large volume of data in an agile and accessible format. Quality of information in healthcare systems is influenced by the system’s usability and the training of professionals, which, in turn, is linked to their ability to exercise clinical reasoning.
LILACS, Cordeiro *et al*., Brazil, 2019^(11)^	Descriptive, exploratory, qualitative study	Explore nurses’ opinions on the use of EPR as a tool for SNC in the emergency department.	It contributes to the SNC implementation in the emergency department, allowing recorded information to be easily accessed at any time.
LILACS, Rosa *et al.,* Brazil, 2019^(43)^	Mixed methods study	Assess the usability of the SNC module of the Brazilian Health System’s Healthcare Management System by ICU nurses in hospitals owned by the Paraná State Health Department.	It facilitates management practices, clinical assessment, diagnoses, and nursing interventions, due to the system’s logical structure and efficient information processing. It contributes to knowledge development and improved quality of patient care.

*EHR - Electronic Health Record; EPR - Electronic Patient Record; NP - Nursing Process; ICU - Intensive Care Unit; USA - United States of America; SNC - Systematization of Nursing Care; MEDLINE - Medical Literature Analysis and Retrieval System Online; LILACS - Latin American and Caribbean Literature in Health Sciences.*

**Chart 5:** Summary of articles included in class 4 of the *Interface de R pour les Analyses Multidimensionnelles de Textes et de Questionnaires* for the final sample of the scoping review, Londrina, Paraná, Brazil, 2024

Database, first author, country and year	Study design	Objectives	Applicability of EHR in NP
MEDLINE, Nantschev *et al*., Austria, 2022^(44)^	Representative quantitative research	Explore the challenges of secondary use of data routinely collected by nursing staff in Austrian acute care hospitals.	Training nursing staff and management to collect and process electronic records, generating secondary data for nursing-sensitive analyses.
MEDLINE, Macieira *et al*., USA, 2021^(45)^	Quantitative convergent parallel mixed methods research	Describe a new methodology for transforming complex nursing care plan data into meaningful variables for assessing the impact of nursing care.	Using an artificial intelligence platform, raw data recorded in EPR are transformed into representative constructs that can be used in research and specialized nursing practice.
Scopus, Luan, *et al*., China, 2023^(46)^	Bibliometric analysis	Explore the application of EHRs in nursing and determine the current status of research and critical areas.	The research encourages nurses to use EHRs to support clinical work and scientific research.
Scopus, Liljamo *et al*., Finland, 2020^(47)^	Retrospective registry study	Examine the relationship between coded nursing diagnoses, nursing interventions, and nursing intensity, and discuss the possibilities for reusing nursing data for workload planning.	There are possibilities for reusing coded nursing data for administrative and resource planning purposes.

*EHR - Electronic Health Record; EPR - Electronic Patient Record; NP - Nursing Process; USA - United States of America; MEDLINE - Medical Literature Analysis and Retrieval System Online.*

The articles belonging to class 1, 27.27% of the total found, deal with the adequacy of filling out computerized documents and forms in NP^(17-25)^.

In class 2, there are articles that permeate computerized documentation in NP as a tool to support clinical decision-making and intervention, in order to subsidize safe care, corresponding to 42.4%^(12,26-38)^.

Belonging to class 3, there are 18% of articles, which address the assessment of healthcare professionals on the usability of EHR in NP and improvements in quality of the daily work process^(11,39-43)^.

To a lesser extent (12%), articles in class 4 elucidate the potential of EHR to generate secondary data from information in NP to support the scientific field with the production of different types of research^(44-47)^.

The similarity analysis performed by IRAMUTEQ identified coincidences between words, and its results indicate connections between them. From this result, a semantic range of the most frequent words was generated. It was found that “nursing” and “care” organized all other terms in the *corpus*. The word “care” branched closely with the words “information” and “decision” and distally with the words “research”, “efficacy”, and “improvement”.

Another important branch is “record”, from which the word “standardize” derives. From “process” comes the word “software”. From “electronic”, the words “paper” and “need” emerged. Other branches, such as “system”, “result”, “health”, “diagnosis”, and “documentation”, were also relevant, giving rise to the term “digital”. In more distant but no less important branches, the word “nurse” emerged, giving rise to the words “environment” and “quality”, and the word “patient”, connected to “safety”.

## DISCUSSION

Brazil has made progress towards modernizing its healthcare systems, with emphasis on incorporating EHRs as a strategy to improve care and ensure patient safety^(48)^. The predominance of national publications in this review reaffirms this movement, reflecting the growing interest of Brazilian nursing in NP computerization, agreeing with Silva *et al*.^(49)^.

Health technology is an instrument that promotes agility, security and speed in accessing information^(50)^. In this context, the similarity analysis carried out in IRAMUTEQ highlighted the terms “nursing”, “care” and “information” as central, reinforcing the role of EHR in the organization of care and in the appreciation of nurses’ clinical reasoning^(31)^. These systems structure decision-making, optimize care time, and promote communication among teams, essential elements for evidence-based practice^(51)^.

This semantic centrality reflects the transformative role of EHR in empowering nurses’ clinical practice. More than simply recording actions, EHR allows nurses to act as producers and managers of qualified information, promoting reflective and responsive practice^(50)^. This new paradigm requires digital skills and sophisticated clinical reasoning, which must be encouraged from academic training onwards^(51)^.

The final sample showed considerable methodological heterogeneity, comprising studies with diverse designs, including quantitative studies (cohort, cross-sectional, retrospective, record analysis, and bibliometric analysis), qualitative studies, mixed methods studies, and integrative reviews. This diversity reflects both the complexity of the topic and the different contexts and stages of scientific development related to the applicability of EHRs in NP.

In class 1, studies showed that EHRs improve the documentation stage of NP by ensuring standardization and integrity of information. However, challenges such as duplicate records and system underutilization persist, often associated with poor training and limited institutional recognition of computerized NP^(18,19)^.

The existence of flaws in the academic training of nursing professionals results in the use of NP in a superficial and fragmented manner so that nurses do not take ownership of this tool, reducing its potential as a clinical tool^(21)^.

In their study, Kilic and Korkmaz^(18)^ observed that nursing data related to medical treatment were recorded in EPR, while nursing data remained in paper records. This finding elucidates the cultural hierarchy that exists between the two professions, perpetuating the idea that medical interventions are more significant than nursing interventions^(41,52)^.

However, nurses’ role is fundamental for developing activities related to care organization, nursing team supervision and coordination, in addition to multidisciplinary coordination^(42)^. Therefore, the appropriation of scientific knowledge and speeches based on clinical reasoning provide the appreciation of clinical nursing practices and the strengthening of the image of nurses^(53)^.

Within class 2, the articles pointed out that EHR contribute directly to care qualification, time optimization and improvement of the experience of professionals with records^(26,28)^. Additionally, it improves nurses’ experience with the system by integrating protocols and risk scales, as well as allowing real-time access to clinical history, resulting in higher quality nursing records^(26-33)^. These findings also highlighted EHRs as instruments to support clinical decision-making, contributing to safer and more effective care^(28)^.

Igarashi *et al*.^(6)^ associated the use of EHRs with the reduction of medication errors by providing clinical decision support and optimizing workflows and institutional organization. Therefore, computerization strengthens the visibility and efficiency of nurses’ decision-making processes, which are essential for achieving high levels of patient safety^(30,32-33)^. Hence, electronic records, as a legal requirement, must be used continuously and in a structured manner, contributing to care organization and reducing work overload^(43)^.

The articles belonging to class 3 addressed that the adoption of electronic systems in nursing contributes to reducing workload and improving professional satisfaction by facilitating the recording of data collected during care^(11,39-43)^. The authors reinforce the importance of standardizing records, enabling the rapid collection of large amounts of data, facilitating the application of NP and clinical reasoning in emergency situations^(8,42)^.

However, unintuitive interfaces and usability issues compromise the effectiveness of registrations, making the process more time-consuming and prone to errors. This highlights the importance of user-centric systems with continuing education^(54)^.

In class 4, the potential of EHRs was identified as a source of secondary data to support the scientific field with the production of different types of research. The information derived from computerized EHRs promotes improvements in care practice and clinical decision-making, in addition to contributing to the allocation of personnel and material resources^(44-46)^.

Within clinical research, the use of EHRs covers several applications, from supporting clinical investigations to randomized clinical trials based entirely on EPR information^(55)^. Auditors, for instance, value the ability to monitor activities on the front lines of care, where clinical decisions and workflows directly influence patient outcomes^(6)^.

It is important to note that EHRs, as a primary source of information, in research contexts involving human beings, must be based on ethical principles, including obtaining patients’ consent for the use of their medical records^(56)^.

Thus, the findings of this review indicate that the implementation of EHR in NP has a positive impact on the organization of care, information security, multiprofessional communication and the visibility of the nursing work process^(45,57)^. When properly implemented, these systems reduce workload, increase job satisfaction, and promote safe, efficient, and evidence-based clinical practice. NP computerization thus emerges as an essential tool for strengthening nursing and continuously improving healthcare^(51-53)^.

The methodological diversity observed in the studies included in this review translates into a varied composition of designs, with a predominance of qualitative research, descriptive and exploratory studies, technological development studies, and documentary analyses. These types of studies are fundamental for understanding processes, perceptions, and experiences, but according to traditional hierarchies of evidence, they are classified as lower in terms of robustness when compared to clinical trials or robust analytical observational studies^(58)^.

Therefore, the interpretation of the findings must consider these methodological limitations without, however, disregarding the relevance of the knowledge produced to date, which is essential to support the development and consolidation of the use of EHRs in hospital NP.

### Study limitations

Since this is a field still under development, there is a gap in the production of studies with high methodological rigor and a higher level of evidence, which reinforces the need to develop future investigations that use more robust designs, capable of objectively assessing the effects of EHRs on clinical practice, patient safety, and the efficiency of healthcare services.

Although some quantitative studies have been found, such as medical record analyses and retrospective studies, they are still not sufficient to establish causal relationships or broadly measure the impacts of EHRs in NP on clinical or organizational outcomes.

Furthermore, the heterogeneity of included studies made it difficult to synthesize the results, since different contexts and electronic health record systems can influence the findings. Furthermore, the methodological quality of primary studies varied significantly, compromising the reliability of the results obtained.

Finally, it is essential to carefully assess the outcomes of each study to avoid inappropriate generalizations, considering that the applicability of electronic medical records may vary among different healthcare institutions and levels of available resources.

### Contributions to health

NP records deserve attention, as they act as a tool to prove nursing care execution, as well as risk management, contributing to service quality and patient safety.

The applicability of EHRs to NP in hospital settings streamlines the work process due to the ease of technology and reduction in costs of physical medical records. Furthermore, since professional overload is one of the impediments to NP implementation, investments in software can increase nursing team satisfaction and improve systematization of care for NP uniformity.

Through training professionals in new technologies, in addition to increasing productivity and quality, EHRs’ contribution permeates the culture of innovation, enabling nurses to appropriate work tools and clinical reasoning, attributing greater value to their work.

## CONCLUSIONS

This study highlights the applicability of EHRs in NP, highlighting their role in improving quality of care and patient safety. However, the results indicate that the effectiveness of EHRs is influenced by contextual factors, such as institutional resources and integration with other health technologies. The heterogeneity of studies and variations in methodological quality indicate the need for caution when interpreting the findings, and it is essential to adapt practices to different institutional settings.

## Data Availability

The research data are available within the article.
