# Supplementary material for: Applicability of electronic health records in the hospital Nursing Process: a scoping review
Source: Rev Bras Enferm. 2025 Dec 8;78(Suppl 3):e20240520. doi: 10.1590/0034-7167-2024-0520 (PMC12707854; doi:10.1590/0034-7167-2024-0520)
Supplement: Translation

# Translation: Aplicabilidade dos registros eletrônicos de saúde no Processo de Enfermagem hospitalar: revisão de escopo

**DOI:** 10.1590/0034-7167-2024-0520pt

**Authors:** Isabela Rodrigues da Silva, Elisana Agatha Iakmiu Camargo Cabulon, Fernanda Santiago Santos Mendonça, Flavia Mendonça da Silva Oussaki, Maria do Carmo Fernandez Lourenço Haddad

**Affiliations:** I Universidade Estadual de Londrina. Londrina, Paraná, Brasil; II Universidade Estadual de Maringá. Maringá, Paraná, Brasil; III Universidade Federal de São Paulo. São Paulo, São Paulo, Brasil

**Keywords:** Processo de Enfermagem, Cuidado de Enfermagem, Informática em Enfermagem, Registros Eletrônicos de Saúde, Registros Médicos.

## Abstract

**Objetivos:**

sintetizar as evidências científicas sobre a aplicabilidade dos Registros Eletrônicos de Saúde no Processo de Enfermagem em hospitais.

**Métodos:**

revisão de escopo seguindo as diretrizes do *Preferred Reporting Items for Systematic Reviews and Meta-Analyses*, com buscas em seis bases de dados eletrônicas, restringindo-se a publicações em português, inglês e espanhol de 2019 a 2024.

**Resultados:**

encontraram-se 2.789 resultados, com 33 artigos incluídos na pesquisa e classificados em quatro categorias temáticas. Os países com mais publicações incluem Brasil, Estados Unidos e China, com destaque para o ano de 2021.

**Conclusões:**

os registros do Processo de Enfermagem hospitalar são cruciais para comprovar a assistência e gerenciar riscos, melhorando a qualidade e segurança do paciente. A implementação dos Registros Eletrônicos de Saúde otimiza o trabalho e reduz custos, aliviando a sobrecarga da equipe. Além de subsidiar a tomada de decisão clínica, tais registros também permitem gerar dados secundários que impulsionam pesquisas científicas.

## INTRODUÇÃO

Introduzido no Brasil por Wanda Horta, o conceito Processo de Enfermagem (PE) permitiu que a assistência prestada ao indivíduo adquirisse caráter formalizado e sistematizado^(1)^. Esse conceito diz respeito à organização do trabalho do enfermeiro, no sentido de garantir qualidade à assistência prestada^(2)^. Nesse contexto, inovações tecnológicas, como os Registros Eletrônicos de Saúde (RES), surgem como ferramentas estratégicas para potencializar a operacionalização do PE nas instituições de saúde^(3)^.

No contexto brasileiro, a implementação de sistemas digitais para a gestão das informações de saúde avançou ao longo dos anos. A partir de 2013, com iniciativas como a Política Nacional de Atenção Básica e a Rede de Atenção à Saúde, o uso de sistemas informatizados, como o Prontuário Eletrônico do Paciente (PEP), começou a se expandir, com o objetivo de melhorar a qualidade da assistência e a gestão das informações clínicas^(4)^.

Em 2024, o Conselho Federal de Enfermagem publicou a Resolução nº 736/2024, determinando a implementação do PE em todo contexto socioambiental no qual ocorram cuidados prestados pela enfermagem. Devido às novas perspectivas da profissão, a delimitação de seu escopo de aplicação na prática se faz necessária, adequando o PE enquanto ferramenta norteadora do raciocínio clínico do enfermeiro no processo de trabalho^(5)^.

Por se tratar de documentação legal e ética do exercício da enfermagem, é imprescindível o registro formal do PE no prontuário do paciente, seja físico ou eletrônico^(5)^. Sob essa ótica, a transformação digital nos serviços de saúde tem promovido mudanças significativas na prática da enfermagem, especialmente com a implantação dos RES em ambientes hospitalares^(6)^.

Estudos apontam que, sem os registros do PE, não é possível resgatar, de forma sistemática, o processo de trabalho executado pela enfermagem, o que reforça estratégias eficazes para melhorar a qualidade dos registros clínicos nos serviços de saúde^(2)^. Tais estratégias frequentemente incluem inovações no campo da comunicação e informação, as quais desempenham papel fundamental no aprimoramento da captação de dados e na melhoria do cuidado prestado^(7)^.

Nesse sentido, tecnologias como o RES têm como objetivo otimizar a captura, o acesso e compartilhamento de informações clínicas, impactando diretamente a qualidade e segurança do atendimento^(8)^. O uso do RES facilita o acesso rápido a dados atualizados do paciente, apoia a tomada de decisões clínicas fundamentadas, promove a padronização e a legibilidade dos registros, e contribui significativamente para a eficiência e segurança da assistência prestada^(6)^.

Estudo demonstrou que mais da metade das tecnologias de apoio aos RES foi relacionada à construção de *softwares* voltados ao ensino-aprendizagem de enfermeiros^(9)^. De forma geral, tais tecnologias permitem melhor controle de documentos e arquivamento das informações relativas à assistência, contribuindo para a melhoria dos processos de trabalho e qualificando a assistência prestada ao paciente^(10)^.

Especificamente no ambiente hospitalar, destaca-se o uso do PEP como RES e ferramenta tecnológica de comunicação cuja finalidade visa gerir eficiência e eficácia na gestão hospitalar^(11)^. O PEP constitui-se em estratégia segura de registro e armazenamento de informações do paciente que podem ser compartilhadas entre a equipe de saúde, evitando eventos adversos^(12)^.

No entanto, o uso do PEP no PE enfrenta diversas fragilidades que impactam a qualidade do cuidado e eficiência do trabalho dos profissionais^(8)^. A resistência dos profissionais de saúde à mudança, aliada à falta de capacitação adequada, dificulta a adaptação ao sistema, resultando em registros imprecisos ou incompletos^(13)^. Ademais, a segurança e privacidade dos dados também causam preocupações constantes, pois o uso inadequado do PEP pode expor informações sensíveis a riscos cibernéticos^(14)^.

Portanto, torna-se imprescindível investigar e propor soluções que fortaleçam a prática do PE, promovendo sua integração nas rotinas de trabalho dos enfermeiros e melhorando a qualidade dos registros clínicos, o que é fundamental para a segurança do paciente e eficiência na gestão hospitalar^(15)^.

## OBJETIVOS

Sintetizar as evidências científicas da aplicabilidade dos RES no PE em ambiente hospitalar e como esses achados podem contribuir para a prática de enfermagem.

## MÉTODOS

### Desenho, período e local do estudo

Trata-se de revisão no formato de revisão de escopo pautada pelo referencial teórico-metodológico do manual de revisão de escopo do JBI, seguindo os passos determinados pelo *checklist Preferred Reporting Items for Systematic Reviews and Meta-Analyses for Scoping Reviews*. Além de rastrear os estudos da temática de interesse, esse tipo de pesquisa examina a extensão e potencialidades que os achados possuem em tornar-se revisões sistemáticas^(16)^. O protocolo da presente revisão foi previamente registrado na plataforma *Open Science Framework* sob o endereço: https://osf.io/86bpn/.

Realizou-se recorte temporal dos anos de 2019 a 2024 e linguístico, incluindo os estudos em português, inglês e espanhol, devido à grande quantidade de resultados, embora recomendação metodológico do JBI seja mais abrangente.

Com a finalidade de alcançar diversos tipos de estudo, foram utilizadas as seguintes bases de dados eletrônicas: *Medical Literature Analysis and Retrieval System Online* (MEDLINE via PubMed); Literatura Latino-Americana e do Caribe em Ciências da Saúde (LILACS); *Web of Science* (WoS); *Excerpta Medica dataBASE* (Embase); Elsevier (Scopus); e *Cumulative Index to Nursing and Allied Health Literature* (CINAHL). Estudos duplicados e não acessíveis na íntegra em bases abertas ou restritas não foram incluídos no presente estudo.

Em consonância com os pressupostos metodológicos das revisões de escopo, priorizaram-se a abrangência e relevância dos estudos para a pergunta de pesquisa - voltada à aplicabilidade dos RES no PE em ambiente hospitalar -, independentemente de delineamento ou rigor metodológico individual. Essa abordagem possibilitou uma análise ampla e descritiva da literatura existente, adequada aos objetivos exploratórios da revisão.

### População ou amostra; critérios de inclusão e exclusão

Empregou-se a estratégia *Population, Concept and Context* (PCC), sendo P aplicabilidade do PEP no desenvolvimento do PE, C, quantidade e conteúdo das evidências científicas publicadas sobre o uso do PEP no PE, e C, ambiente hospitalar. A partir disso, chegou-se à seguinte pergunta de pesquisa: quais as evidências científicas da aplicabilidade do RES no PE em ambiente hospitalar? Foi possível mapear e explorar a literatura científica acerca da temática escolhida, permitindo identificar como os estudos foram conduzidos^(8)^.

Incluíram-se estudos dos tipos experimental, quase-experimental (ensaios clínicos randomizados e não randomizados), observacionais (coorte, caso-controle e transversais) e qualitativo. Também fizeram parte desta revisão relatos de caso, protocolos, diretrizes de prática clínicas, todos os tipos de síntese de evidências, teses, dissertações e trabalhos de conclusão de curso.

Artigos com dados incompletos, cartas, editoriais e estudos que, após três tentativas consecutivas de contato com os autores, não tiveram acesso disponível na íntegra não foram incluídos nesta revisão de escopo.

### Protocolo do estudo

A estratégia de busca se adaptou para cada base de dados, de acordo com os descritores recomendados em cada uma delas. Os termos foram combinados com os operadores booleanos “*AND*” e “*OR*” nas bases de dados Biblioteca Virtual em Saúde e Embase, a fim de identificar termos de busca referentes à temática da pesquisa, por meio de estudos considerados relevantes. Com auxílio das plataformas Descritores em Ciências da Saúde (DeCS) e *Medical Subject Headings* (MeSH), as estratégias utilizadas em cada base de dados encontram-se descritas no Quadro 1.

**Quadro 1:** Estratégias de buscas nas plataformas Descritores em Ciências da Saúde e *Medical Subject Headings*, 2024

Base de dados	Tipo	Estratégias de busca
MEDLINE (via PubMed)	MeSH	(((“*Nursing Process*”) *OR* (“Nursing Care”)) *AND* (“*Nursing Informatics*”)) *OR* (“*Electronic Health Records*”)
WoS	MeSH	(“*Nursing Process*”) *OR* (“*Patient Care Planning*”) *AND* (“*Nursing Informatics*”) OR (“*Electronic Health Records*”)
Scopus	MeSH	(“*Nursing Process*”) *OR* (“*Patient Care Planning*”) *AND* (“*Nursing Informatics*”) OR (“*Electronic Health Records*”)
LILACS	DeCS	(“Processo de Enfermagem”) OR (“Cuidados de Enfermagem”) AND (“Informática em Enfermagem”) OR (“Registros Eletrônicos de Saúde”)
Embase	*Emtree* *Thesaurus*	(*‘Nursing Process’ OR ‘Nursing Care’*) *AND* (*‘Nursing Informatics’ OR ‘Medical Record*’)
CINAHL	MeSH	(“*Nursing Process*” *OR* “Nursing Care”) *AND* (“*Nursing Informatics*” *OR* “*Nursing Records*” *OR* “*Electronic Health Record*”)

*WoS - Web of Science; DeCS - Descritores em Ciências da Saúde; MeSH - Medical Subject Headings; LILACS - Literatura Latino-Americana e do Caribe em Ciências da Saúde; MEDLINE - Medical Literature Analysis and Retrieval System Online; CINAHL - Cumulative Index to Nursing and Allied Health Literature; Embase - Excerpta Medica dataBASE.*

### Análise dos resultados e estatística

Os estudos foram agrupados e exportados para a plataforma Rayyan QCRI, com o objetivo de realizar a triagem e seleção de forma cega e imparcial entre dois revisores independentes, após a exclusão automática dos estudos duplicados. Essa ferramenta permitiu a realização da triagem sem que os avaliadores tivessem acesso às decisões um do outro, assegurando a neutralidade do processo. Em casos de discordância entre os avaliadores quanto à inclusão ou exclusão dos estudos, um terceiro revisor foi acionado para resolução do impasse.

A análise inicial foi conduzida por meio da leitura dos títulos e resumos, identificando aqueles que apresentavam aderência à pergunta de pesquisa. Os estudos selecionados foram, então, lidos na íntegra para confirmação de sua elegibilidade, e os que atenderam aos critérios estabelecidos foram sintetizados de forma descritiva, organizados em tabelas e acompanhados de um resumo narrativo, conforme as etapas metodológicas preconizadas pelo manual de revisão de escopo do JBI.

Ainda conforme recomendações do JBI, o mapeamento dos dados ocorreu com extração de dados, contemplando base de dados, autoria, país, ano, objetivo e aplicabilidade do RES no PE. Tais informações alimentaram a planilha eletrônica do *Microsoft Office Excel* ^®^ 2019, possibilitando a extração de dados para construção da síntese.

Como resultado da seleção dos estudos, empregou-se um fluxograma de todas as etapas e com os respectivos resultados. Além disso, os dados foram organizados em quadros descritivos, a fim de facilitar a compreensão e colaborar com a construção da discussão dos resultados.

Juntamente com as tabulações dos resultados, o *software Interface de R pour les Analyses Multidimensionnelles de Textes et de Questionnaires* (IRAMUTEQ) foi utilizado para agrupar os resultados, o qual gerou um dendrograma com classes, de modo a destacar as situações relevantes e a similaridade entre os achados. Possibilitou-se, assim, a descrição narrativa dos achados, além de direcionamento da correlação com o objetivo e a pergunta da revisão.

## RESULTADOS

Foram encontradas 2.789 publicações, das quais 171 foram excluídas devido à duplicidade, restando 2.618 estudos para leitura dos títulos e resumos, dos quais 59 foram selecionados para leitura na íntegra. Destes, 33 foram incluídos na revisão por responderem à pergunta de pesquisa e serem condizentes com os critérios de elegibilidade (Figura 1).

**Figura 1:** Fluxograma do processo de seleção de estudos conforme diretrizes do *Preferred Reporting Items for Systematic Reviews and Meta-Analyses for Scoping Reviews*

Do total de artigos incluídos nesta revisão de escopo, 13 foram publicados no Brasil, quatro, nos Estados Unidos, quatro, na China, dois, na Finlândia, dois, na Turquia e dois, na Austrália. Irã, Suíça, Canadá, México e Itália publicaram um artigo.

Utilizou-se o *software* IRAMUTEQ para análise de similaridade entre os estudos incluídos, fornecendo quatro classes de acordo com a estatística do *corpus* textual do programa, com aproveitamento de 75%.

A partir do *corpus*, os segmentos de texto apresentados em cada classe foram obtidos das palavras estatisticamente significativas, permitindo que a análise qualitativa dos dados fosse realizada. O processamento do *corpus* foi realizado em sete segundos, sendo classificadas 966 formas (*Unité Contextuelle d’Enregistrement*), das quais 698 foram aproveitadas, resultando 75% do total do *corpus*, considerado como um aproveitamento adequado.

Pela leitura das palavras em destaque e da sua inserção nos segmentos de texto, foi possível alcançar os objetivos da pesquisa de identificação das evidências científicas da aplicabilidade do RES no PE no ambiente hospitalar. A seguir, os Quadros 2, 3, 4 e 5 apresentam a síntese das informações referentes aos estudos incluídos nesta revisão de escopo, categorizados de acordo com as classes organizadas pelo IRAMUTEQ.

**Quadro 2:** Síntese dos artigos incluídos na classe 1 do *Interface de R pour les Analyses Multidimensionnelles de Textes et de Questionnaires* referente à amostra final da revisão de escopo, Londrina, Paraná, Brasil, 2024

Base de dados, primeiro autor, país e ano	Tipo de estudo	Objetivos	Aplicabilidade do RES no PE
MEDLINE, Karp *et al*., EUA, 2019^(17)^	Estudo de coorte prospectivo	Avaliar se os cronômetros e registros de eventos do sistema de PEP podem medir a eficiência e qualidade de um processo clínico.	Eficiência e qualidade de processo clínico por meio da otimização do tempo depreendido nos registros de enfermagem.
CINAHL, Ozduyan Kilic e Korkmaz Turquia, 2023^(18)^	Revisão de literatura	Avaliar até que ponto os enfermeiros na Turquia usam sistemas de RES/sistemas de informação hospitalar e quais dados são registrados.	Dados de enfermagem relacionados ao tratamento médico foram documentados mais frequentemente eletronicamente, e os relacionados ao PE foram mantidos em papel.
CINAHL, Sanchez *et al*., Brasil, 2019^(19)^	Estudo descritivo qualitativo, tipo estudo de caso	Avaliar o registro dos enfermeiros nos prontuários eletrônico do paciente e convencional, com vista à melhoria da qualidade do cuidado.	Melhoria da coordenação e qualidade do cuidado e segurança do paciente.
CINAHL, Menezes e Lopes Neto, Brasil, 2019^(20)^	Estudo de produção tecnológica, abordagem qualitativa	Relatar o desenvolvimento de *software* para Sistematização da Assistência de Enfermagem em doenças tropicais e infecciosas.	O uso do *software* melhorou o desempenho da prática assistencial da enfermagem, comunicação da equipe e agilidade na tomada de decisão.
CINAHL, Faruch *et al*., Brasil, 2021^(21)^	Estudo transversal, retrospectivo, documental, com abordagem quantitativa dos dados	Avaliar a implementação do PE em hospital universitário.	O uso de *softwares* é facilitador da implementação do PE, providenciando suporte ao fornecer dados para avaliação de diagnósticos, intervenções e registro de informações relevantes sobre a equipe e os pacientes.
MEDLINE, Macieira *et al*., EUA, 2019^(22)^	Revisão sistemática	Apresentar os resultados de revisão sistemática de estudos envolvendo análises secundárias de dados codificados com terminologias padronizadas de enfermagem recuperadas de RES.	O RES melhora a eficiência, padroniza a documentação dos cuidados de enfermagem, gera dados consistentes, facilita o compartilhamento de informações e permite análise para melhorar a prática clínica e os resultados dos pacientes.
MEDLINE, Yilmaztturk *et al*., Turquia, 2023^(23)^	Estudo observacional	Medir o efeito da digitalização de prontuários mantidos em papel em UTI na economia de tempo e no consumo de papel.	Há economia de tempo e custo no ambiente em ambiente hospitalar.
Scopus, Fritzen *et al*., Brasil, 2023^(24)^	Estudo experimental	Implementar, em *software* de gestão em saúde, registros eletrônicos do PE perioperatório e as etapas do transoperatório e pós-operatório imediato, além dos diagnósticos de enfermagem baseados na NANDA-I.	Registros eletrônicos do PE perioperatório possibilitam o gerenciamento de saúde, contribuindo para aperfeiçoamento das práticas assistenciais.
Embase, Chen *et al*., China, 2021^(25)^	Revisão retrospectiva de prontuários	Investigar as características clínicas de constipação e seus fatores de risco em pacientes com hemorragia intracerebral pelos registros eletrônicos de cuidados de enfermagem.	Os RES resultaram na identificação de pacientes com constipação.

*RES - Registro Eletrônico de Saúde; PEP - Prontuário Eletrônico do Paciente; PE - Processo de Enfermagem; UTI - Unidade de Terapia Intensiva; NANDA-I - NANDA International; EUA - Estados Unidos da América; Embase; MEDLINE - Medical Literature Analysis and Retrieval System Online; CINAHL - Cumulative Index to Nursing and Allied Health Literature; Embase - Excerpta Medica dataBASE.*

**Quadro 3:** Síntese dos artigos incluídos na classe 2 do *Interface de R pour les Analyses Multidimensionnelles de Textes et de Questionnaires* referente à amostra final da revisão de escopo, Londrina, Paraná, Brasil, 2024

Base de dados, primeiro autor, país e ano	Tipo de estudo	Objetivos	Aplicabilidade do RES no PE
CINAHL, Zhao *et al*., China, 2021^(26)^	Estudo de caso controle	Desenvolver novo sistema para melhorar a prática de enfermagem e garantir a continuidade e integridade nos cuidados de enfermagem à hipertermia.	O sistema desenvolvido avaliou a melhoria da qualidade da assistência de enfermagem, otimizando tempo de atendimento e experiência do sistema pelos enfermeiros, e melhora na qualidade dos registros de enfermagem.
CINAHL, Oliveira *et al*., Brasil, 2021^(27)^	Estudo quantitativo quase-experimental	Comparar a qualidade da documentação do PE em duas versões de um sistema de apoio à decisão clínica.	A integração com sistemas de apoio à decisão clínica facilita a análise e o suporte na tomada de decisão dos profissionais de enfermagem, além de contribuir para a organização das informações, garantindo a continuidade e qualidade dos cuidados prestados ao paciente.
CINAHL, *et al*., Brasil, 2019^(12)^	Estudo metodológico	Descrever a construção e validação de registros eletrônicos do PE em instituição oncológica.	Favorece a tomada de decisão do enfermeiro e qualificação do cuidado através dos registros eletrônicos interligados.
MEDLINE, Akbar *et al*., Austrália, 2021^(28)^	Revisão sistemática	Resumir a literatura sobre sistemas automatizados de apoio à decisão em enfermagem e analisar os seus efeitos na prestação de cuidados e resultados do paciente.	Integração dos sistemas de apoio à decisão, adesão de informações e documentação das intervenções de enfermagem promovem a eficiência e qualidade nos cuidados de saúde.
Scopus, Von Gerich *et al*., Finlândia, 2023^(29)^	Estudo piloto	Explorar o potencial dos RES como uma fonte de informação para avaliar a eficácia dos cuidados de enfermagem.	Os registros eletrônicos são fontes de informação no desenvolvimento de sistemas de apoio à decisão clínica, gestão em saúde e avaliação da efetividade dos cuidados de enfermagem.
Scopus, Franco *et al*., Brasil, 2023^(30)^	Relato de experiência	Descrever o desenvolvimento da informatização de escalas de predição de risco, utilizadas pela enfermagem no prontuário eletrônico, no sistema AGHUse^®^.	As funcionalidades desenvolvidas na informatização das escalas de predição de risco favoreceram a sua operacionalização, refletindo-se positivamente na prática do enfermeiro e na segurança do paciente.
Scopus, Hants *et al*., Austrália, 2023^(31)^	Revisão Sistemática Integrativa	Identificar como o PE foi incorporado aos registros digitais de saúde.	A informatização de todas as etapas do PE possibilita o avanço dos processos de tomada de decisão dos enfermeiros, bem como a visibilidade do seu trabalho.
Scopus, Szydłowska-Pawlak *et al.,* Suíça, 2022^(32)^	Estudo de caso	Formular um plano de cuidados para um recém-nascido com diagnóstico de gastrosquise congênita no pós-operatório, utilizando a Classificação Internacional para a Prática de Enfermagem, que foi incluída no *software* “ADPIECare Dorothea”.	Os RES são ferramentas de apoio à tomada de decisão clínica do enfermeiro. Os dados podem ajudar a otimizar a organização do trabalho para atingir níveis elevados de saúde e segurança do paciente.
Scopus, Wu *et al*., China, 2020^(33)^	Estudo de revisão	Descrever o desafio dos RES e da aplicação do sistema de apoio à decisão de enfermagem baseado no PE.	O sistema de apoio à decisão clínica é amparado pelos registros eletrônicos que os enfermeiros coletam durante a assistência, o que permite melhorar a qualidade e segurança do cuidado.
Scopus, Bail *et al.,* Austrália, 2021^(34)^	Auditoria retrospectiva	Explorar e comparar a documentação do PE para segurança do paciente em dois sistemas de documentação de enfermagem: registros em papel e digitais.	Os sistemas eletrônicos têm potencial para melhorar a captura do raciocínio clínico e do PE.
Scopus, Sanson *et al.,* Itália, 2019^(35)^	Estudo observacional prospectivo	Explorar o poder preditivo independente dos diagnósticos de enfermagem na mortalidade hospitalar dos pacientes e investigar se a inclusão desta variável além dos dados de diagnóstico médico pode melhorar o desempenho das ferramentas de ajuste de risco.	Os diagnósticos de enfermagem têm poder independente na predição da mortalidade hospitalar. A variância explicada nos modelos preditivos melhorou quando os dados eletrônicos de enfermagem foram incluídos além dos dados médicos.
MEDLINE, Burkoski *et al*., Canadá, 2021^(36)^	Estudo descritivo	Descrever o desenvolvimento da abordagem inovadora baseada em registros médicos eletrônicos do *Humber River Hospital* para triagem de pacientes com risco de resultados adversos, usando a Associação de Enfermeiros Registrados de Ontário e diretrizes de melhores práticas.	Os registros eletrônicos facilitam a realização de avaliações e intervenções para prestação de cuidados baseados em evidências. Também criam barreiras para que os enfermeiros alinhem suas ações aos padrões estabelecidos, reduzindo variabilidade da prática de enfermagem.
LILACS, Chen Weiwin *et al.,* México, 2021^(37)^	Estudo transversal, retrospectivo, documental	Avaliar o cumprimento do registro clínico eletrônico de acordo com o modelo do cuidado de enfermagem.	Os registros de enfermagem são essenciais para a tomada de decisão sobre o cuidado apoiado em evidências científicas. Permitem identificar áreas de oportunidade de melhoria, reforçando a adesão aos modelos padronizados de registros de enfermagem.
LILACS, Schamne *et al*., Brasil, 2019^(38)^	Estudo de método misto do tipo exploratório descritivo.	Validar uma proposta para registros de cuidados de enfermagem contendo diagnósticos, resultados esperados, resultados atuais, intervenções e ações de enfermagem para pacientes com deterioração clínica, utilizando o Sistema de Classificação de Cuidados Clínicos.	Contribui para o aprimoramento da tomada de decisão do enfermeiro e na melhoria dos fluxos da informação sobre os cuidados prestados aos pacientes com deterioração clínica.

*RES - Registro Eletrônico de Saúde; PE - Processo de Enfermagem; LILACS - Literatura Latino-Americana e do Caribe em Ciências da Saúde; MEDLINE - Medical Literature Analysis and Retrieval System Online; CINAHL - Cumulative Index to Nursing and Allied Health Literature.*

**Quadro 4:** Síntese dos artigos incluídos na classe 3 do *Interface de R pour les Analyses Multidimensionnelles de Textes et de Questionnaires* referente à amostra final da revisão de escopo, Londrina, Paraná, Brasil, 2024

Base de dados, primeiro autor, país e ano	Tipo de estudo	Objetivos	Aplicabilidade do RES no PE
MEDLINE, Kutney-Lee *et al*., EUA, 2019^(39)^	Estudo de coorte	Examinar os efeitos da adoção abrangente dos RES nos relatórios dos enfermeiros sobre a qualidade do cuidado.	Qualidade do cuidado prestado ao paciente e contribuição com a segurança do paciente.
Scopus, Shafiee *et al*., Irã, 2022^(40)^	Pesquisa metodológica	Descrever o processo de concepção e avaliação do conteúdo de sistema eletrônico de documentação clínica de enfermagem para fornecer relatórios consistentes e unificados.	O sistema eletrônico reduz a quantidade de dados coletados durante o atendimento de enfermagem, o que resulta em maior satisfação profissional pela diminuição da carga de trabalho com essa atividade.
LILACS, Costa *et al*., Brasil, 2020^(41)^	Revisão integrativa	Identificar quais estratégias estão sendo utilizadas na implementação de registros eletrônicos relacionados ao PE nas bases de dados PubMed, Scopus e *Web of Science*.	Sistemas informatizados são amplamente adotados na saúde, exigindo que enfermeiros se capacitem para padronizar os registros de enfermagem, necessitando de constante aprimoramento.
LILACS, Amaral *et al*., Brasil, 2021^(42)^	Estudo transversal	Verificar a opinião de enfermeiros sobre o registro eletrônico de diagnósticos e de disciplinas de enfermagem em sistema informatizado no âmbito hospitalar.	A informatização gera um grande volume de dados de forma ágil e acessível. A qualidade da informação em sistemas de saúde é influenciada pela usabilidade do sistema e capacitação dos profissionais que, por sua vez, está ligada à competência em exercer o raciocínio clínico.
LILACS, Cordeiro *et al*., Brasil, 2019^(11)^	Estudo descritivo, exploratório, qualitativo	Desvelar a opinião dos enfermeiros sobre o uso do PEP como ferramenta para SAE no setor de emergência.	Contribui para a realização da SAE no setor de emergência, permitindo que as informações registradas sejam facilmente acessadas a qualquer momento.
LILACS, Rosa *et al.,* Brasil, 2019^(43)^	Estudo de métodos mistos	Avaliar a usabilidade do módulo de SAE do Sistema de Gestão da Assistência de Saúde do Sistema Único de Saúde por enfermeiros de UTI em hospitais próprios da Secretaria Estadual de Saúde do Paraná.	Facilita práticas de gerenciamento, avaliação clínica, diagnósticos e intervenções de enfermagem, devido à estrutura lógica do sistema e ao processamento eficiente das informações. Contribui para a construção do conhecimento e melhoria da qualidade no cuidado prestado aos pacientes.

*RES - Registro Eletrônico de Saúde; PEP - Prontuário Eletrônico do Paciente; PE - Processo de Enfermagem; UTI - Unidade de Terapia Intensiva; EUA - Estados Unidos da América; SAE - Sistematização da Assistência de Enfermagem; MEDLINE - Medical Literature Analysis and Retrieval System Online; LILACS - Literatura Latino-Americana e do Caribe em Ciências da Saúde.*

**Quadro 5:** Síntese dos artigos incluídos na classe 4 do *Interface de R pour les Analyses Multidimensionnelles de Textes et de Questionnaires* referente à amostra final da revisão de escopo, Londrina, Paraná, Brasil, 2024

Base de dados, primeiro autor, país e ano	Tipo de estudo	Objetivos	Aplicabilidade do RES no PE
MEDLINE, Nantschev *et al*., Áustria, 2022^(44)^	Pesquisa quantitativa representativa	Explorar os desafios do uso secundário de dados coletados rotineiramente pela equipe de enfermagem em hospitais austríacos de cuidados intensivos.	Capacitar equipe e gerência de enfermagem para coletar e processar registros eletrônicos, gerando dados secundários para análises sensíveis à enfermagem.
MEDLINE, Macieira *et al*., EUA, 2021^(45)^	Pesquisa de métodos mistos paralelos convergentes quantitativo	Descrever uma nova metodologia para transformar dados complexos de planos de cuidados de enfermagem em variáveis ​​significativas para avaliar o impacto dos cuidados de enfermagem.	Através de plataforma de inteligência artificial, os dados brutos registrados no PEP são transformados em construtos representativos que podem ser usados na pesquisa e na prática de enfermagem especializada.
Scopus, Luan, *et al*., China, 2023^(46)^	Análise bibliométrica	Explorar a aplicação de RES na área da enfermagem e determinar o *status* atual da pesquisa e os pontos críticos.	As pesquisas incentivam os enfermeiros a usarem os RES como apoio no trabalho clínico e pesquisas científicas.
Scopus, Liljamo *et al*., Finlândia, 2020^(47)^	Estudo retrospectivo de registro	Verificar qual tipo de relação existe entre diagnósticos de enfermagem codificados, intervenções de enfermagem e intensidade de enfermagem, e discutir as possibilidades de reutilização de dados de enfermagem para planejamento de carga de trabalho.	Existem possibilidades de reutilização de dados de enfermagem codificados para fins administrativos e de planejamento de recursos.

*RES - Registro Eletrônico de Saúde; PEP - Prontuário Eletrônico do Paciente; PE - Processo de Enfermagem; EUA - Estados Unidos da América; MEDLINE - Medical Literature Analysis and Retrieval System Online.*

Os artigos pertencentes à classe 1, 27,27% do total encontrado, tratam da adequação quanto ao preenchimento de documentos e formulários informatizados no PE^(17-25)^.

Na classe 2, estão os artigos que permeiam a documentação informatizada no PE como ferramenta de apoio à tomada de decisão clínica e intervenção, a fim de subsidiar um cuidado seguro, correspondente a 42,4%^(12,26-38)^.

Pertencentes à classe 3, encontram-se 18% dos artigos, os quais abordam a avaliação dos profissionais de saúde sobre a usabilidade do RES no PE e melhorias na qualidade do processo de trabalho diário^(11,39-43)^.

Em menor proporção (12%), os artigos da classe 4 elucidam a potencialidade dos RES em gerar dados secundários a partir das informações do PE para subsidiar o campo científico com a produção de diferentes tipos de pesquisa^(44-47)^.

A análise de similitude realizada pelo IRAMUTEQ possibilitou identificar as coincidências entre as palavras, e seu resultado traz indicações da conexão entre elas. A partir do resultado, gerou-se um leque semântico de palavras mais frequentes. Verificou-se que “enfermagem” e “cuidado” organizaram todos os demais termos do *corpus*. A palavra “cuidado” se ramificou proximamente com as palavras “informação” e “decisão” e distalmente com as palavras “pesquisa”, “eficácia” e “melhoria”.

Outro ramo importante é o “registro”, o qual derivou a palavra “padronizar”. Do “processo” derivou-se a palavra “*software*”. Do ramo “eletrônico”, surgiram as palavras “papel” e “necessidade”. Foram relevantes os demais ramos, como “sistema”, “resultado”, “saúde”, “diagnóstico” e “documentação”, originando o termo “digital”. Em ramos mais distantes, porém não menos importantes, surgiu a palavra “enfermeiro”, dando origem às palavras “ambiente” e “qualidade”, e a palavra “paciente” conectada à “segurança”.

## DISCUSSÃO

O Brasil tem avançado na direção à modernização dos seus sistemas de saúde, com destaque para a incorporação dos RES como estratégia para qualificar o cuidado e garantir a segurança do paciente^(48)^. A predominância de publicações nacionais nesta revisão reafirma esse movimento, refletindo o interesse crescente da enfermagem brasileira na informatização do PE, concordando com Silva *et al*.^(49)^.

A tecnologia de saúde é um instrumento promotor de agilidade, segurança e rapidez no acesso às informações^(50)^. Nesse contexto, a análise de similitude realizada no IRAMUTEQ evidenciou os termos “enfermagem”, “cuidado” e “informação” como centrais, reforçando o papel dos RES na organização do cuidado e na valorização do raciocínio clínico do enfermeiro^(31)^. Esses sistemas estruturam a tomada de decisão, otimizam o tempo assistencial e favorecem a comunicação entre equipes, elementos essenciais à prática baseada em evidências^(51)^.

Essa centralidade semântica reflete o papel transformador do RES no empoderamento da prática clínica do enfermeiro. Mais do que registrar ações, o RES permite que o enfermeiro atue como produtor e gestor de informação qualificada, promovendo uma prática reflexiva e responsiva^(50)^. Esse novo paradigma exige competências digitais e raciocínio clínico sofisticado, que devem ser estimulados desde a formação acadêmica^(51)^.

A amostra final apresentou considerável heterogeneidade metodológica, composta por estudos de delineamentos diversos, como estudos quantitativos (coorte, transversais, retrospectivos, análise de registros e análise bibliométrica), qualitativos, de métodos mistos, além de revisão integrativa. Essa diversidade reflete tanto a complexidade do tema quanto os diferentes contextos e estágios de desenvolvimento científico relacionados à aplicabilidade dos RES no PE.

Na classe 1, os estudos evidenciaram que os RES qualificam a etapa documental do PE ao garantir padronização e integridade da informação. No entanto, desafios como a duplicidade de registros e subutilização do sistema persistem, sendo frequentemente associados à formação deficiente e ao pouco reconhecimento institucional do PE informatizado^(18,19)^.

A existência de falhas na formação acadêmica do profissional de enfermagem traz como consequência a utilização do PE de maneira superficial e fragmentada, de forma que o enfermeiro não se apodere de tal ferramenta, reduzindo seu potencial como ferramenta clínica^(21)^.

Em seu estudo, Kilic e Korkmaz^(18)^ observaram que os dados de enfermagem relacionados ao tratamento médico foram registrados no PEP, enquanto os de enfermagem permaneciam em prontuário de papel. Esta constatação elucida a hierarquização cultural existente entre as duas profissões, perpetuando a ideia de que as intervenções médicas são mais significativas que as da enfermagem^(41,52)^.

Contudo, a atuação do enfermeiro é fundamental por desenvolver atividades relacionadas à organização do cuidado, supervisão e coordenação da equipe de enfermagem, além da articulação multiprofissional^(42)^. Portanto, a apropriação de conhecimentos científicos e falas embasadas no raciocínio clínico fornecem a valorização das práticas clínicas de enfermagem e o fortalecimento da imagem do enfermeiro^(53)^.

Dentro da classe 2, os artigos apontaram que os RES contribuem diretamente para a qualificação da assistência, otimização do tempo e melhoria da experiência dos profissionais com os registros^(26,28)^. Além disso, aprimora a experiência dos enfermeiros com o sistema, ao integrar protocolos e escalas de risco, bem como permitir o acesso em tempo real ao histórico clínico, resultando em registros de enfermagem de maior qualidade^(26-33)^. Tais achados ainda destacaram os RES como instrumentos de apoio à decisão clínica, contribuindo para um cuidado mais seguro e resolutivo^(28)^.

Igarashi *et al*.^(6)^ associaram o emprego dos RES à redução de erros de medicação, ao fornecer suporte à decisão clínica e otimização dos fluxos de trabalho e da organização institucional. Portanto, a informatização fortalece a visibilidade e eficiência dos processos decisórios do enfermeiro, essenciais para alcançar níveis elevados de segurança do paciente^(30,32,33)^. Neste sentido, o registro eletrônico, por ser exigência legal, deve ser utilizado de forma contínua e estruturada, contribuindo com a organização da assistência e diminuindo a sobrecarga de trabalho^(43)^.

Os artigos pertencentes à classe 3 abordaram que a adoção de sistemas eletrônicos na enfermagem contribui para redução da carga de trabalho e melhora da satisfação profissional, ao facilitar o registro de dados coletados durante o atendimento^(11,39-43)^. Os autores reforçam a importância da padronização dos registros, possibilitando uma coleta de grande número de dados de forma rápida, facilitando aplicar o PE e o raciocínio clínico em situações de urgência e emergência^(8,42)^.

Contudo, interfaces pouco intuitivas e problemas de usabilidade comprometem a eficácia dos registros, tornando o processo mais demorado e propenso a falhas. Isso destaca a importância de sistemas centrados no usuário e com treinamento contínuo^(54)^.

Na classe 4, identificou-se o potencial dos RES como fonte de dados secundários para subsidiar o campo científico com a produção de diferentes tipos de pesquisa. As informações advindas do PE informatizado promovem melhorias na prática assistencial e tomada de decisão clínica, além de contribuir para o dimensionamento de pessoas e recursos materiais^(44-46)^.

Dentro da pesquisa clínica, a utilização dos RES abrange várias aplicações, desde o apoio para investigações clínicas até ensaios clínicos randomizados baseados integralmente em informações de PEP^(55)^. Os auditores, por exemplo, valorizam a capacidade de monitorar as atividades na linha de frente da assistência, em que decisões clínicas e fluxos de trabalho influenciam diretamente os resultados para os pacientes^(6)^.

É importante atentar-se que os RES, enquanto fonte primária de informação, em contextos de pesquisa envolvendo seres humanos, devem se fundamentar nos princípios éticos, incluindo a obtenção do consentimento do paciente para o uso de seu prontuário^(56)^.

Nesse sentido, os achados desta revisão indicam que a implementação dos RES no PE tem impacto positivo na organização do cuidado, na segurança da informação, na comunicação multiprofissional e na visibilidade do processo de trabalho da enfermagem^(45,57)^. Quando bem implementados, esses sistemas reduzem a sobrecarga de trabalho, aumentam a satisfação profissional e promovem uma prática clínica segura, eficiente e baseada em evidências. A informatização do PE emerge, assim, como uma ferramenta essencial para o fortalecimento da profissão e melhoria contínua da assistência à saúde^(51-53)^.

A diversidade metodológica observada nos estudos incluídos nesta revisão se traduz em uma composição variada de delineamentos, predominando pesquisas qualitativas, estudos descritivos, exploratórios, estudos de desenvolvimento tecnológico e análises documentais. Esses tipos de estudo são fundamentais para compreender processos, percepções e experiências, porém, segundo hierarquias tradicionais de evidência, são classificados como de nível inferior em termos de robustez, quando comparados a ensaios clínicos ou estudos observacionais analíticos robustos^(58)^.

Portanto, a interpretação dos achados deve considerar essas limitações metodológicas sem, contudo, desconsiderar a relevância do conhecimento produzido até o momento, que é essencial para fundamentar o desenvolvimento e a consolidação do uso dos RES no PE hospitalar.

### Limitações do estudo

Por se tratar de um campo ainda em desenvolvimento, observa-se uma lacuna na produção de estudos com alto rigor metodológico e maior nível de evidência, o que reforça a necessidade de desenvolver investigações futuras que utilizem desenhos mais robustos, capazes de avaliar, de forma objetiva, os efeitos dos RES na prática clínica, na segurança do paciente e na eficiência dos serviços de saúde.

Embora tenham sido encontrados alguns estudos quantitativos, como análises de prontuários e estudos retrospectivos, eles ainda não são suficientes para estabelecer relações causais ou mensurar, de forma ampla, os impactos dos RES no PE sobre desfechos clínicos ou organizacionais.

Ademais, a heterogeneidade dos estudos incluídos dificultou a síntese dos resultados, uma vez que diferentes contextos e sistemas de prontuários eletrônicos podem influenciar os achados. Somou-se a isso a qualidade metodológica dos estudos primários que variaram significativamente, o que compromete a confiabilidade dos resultados obtidos.

Por fim, é fundamental avaliar cautelosamente os desfechos de cada estudo para evitar generalizações inadequadas, considerando que a aplicabilidade do prontuário eletrônico pode variar entre diferentes instituições de saúde e níveis de recursos disponíveis.

### Contribuições para a área da saúde

Os registros do PE merecem atenção, pois atuam como ferramenta comprobatória da execução da assistência de enfermagem, bem como de gerenciamento de riscos, colaborando com a qualidade do serviço e segurança do paciente.

A aplicabilidade do RES no PE no ambiente hospitalar agiliza o processo de trabalho devido à facilidade fornecida com a tecnologia e diminuição dos custos com prontuários físicos. Ademais, como a sobrecarga profissional está como um dos impeditivos da execução do PE, investimentos em *softwares* podem corroborar a satisfação da equipe em enfermagem, bem como melhorar a sistematização da assistência para uniformidade do PE.

Por meio de capacitações dos profissionais em novas tecnologias, além do aumento da produtividade e qualidade, a contribuição dos RES permeia a cultura da inovação, possibilitando ao enfermeiro apropriação das ferramentas de trabalho e raciocínio clínico, atribuindo maior valor ao seu trabalho.

## CONCLUSÕES

Este estudo evidencia a aplicabilidade dos RES no PE, destacando seu papel na melhoria da qualidade da assistência e segurança do paciente. Contudo, os resultados apontam que a eficácia dos RES é influenciada por fatores contextuais, como recursos institucionais e integração com outras tecnologias de saúde. A heterogeneidade dos estudos e variações na qualidade metodológica indicam a necessidade de cautela ao interpretar os achados, sendo essencial adaptar as práticas aos diferentes cenários institucionais.

## Data Availability

Os dados da pesquisa estão disponíveis no corpo do artigo.
